# Ultrasound-sensitive siRNA-loaded nanobubbles fabrication and antagonism in drug resistance for NSCLC

**DOI:** 10.1080/10717544.2021.2021321

**Published:** 2021-12-29

**Authors:** Chunhong Su, XiaoJun Ren, Fang Yang, Bin Li, Hao Wu, Hui Li, Fang Nie

**Affiliations:** a Department of Ultrasound Diagnosis, Lanzhou University Second Hospital, Lanzhou, China; b Department of Pain, Lanzhou University Second Hospital, Lanzhou, China; c Department of Pediatric Orthopedics, Lanzhou University Second Hospital, Lanzhou, China; d State Key Laboratory of Bioelectronics, Jiangsu Key Laboratory for Biomaterials and Devices, School of Biological Sciences and Medical Engineering, Southeast University, Nanjing, China

**Keywords:** Targeted delivery, ultrasound irradiation, nanobubbles, drug resistance, ultrasound image

## Abstract

Due to the lack of safe, effective, and gene-targeted delivery technology. In this study, we have prepared nanobubbles loaded PDLIM5 siRNA (PDLIM5siRNA-NBs) to investigate the transfection efficiency and their antagonism in drug resistance in combination with ultrasound irradiation for non-small-cell lung cancer (NSCLC). Research results show that the PDLIM5 siRNA are effectively bound to the shell of NBs with a mean diameter of 191.6 ± 0.50 nm and a Zeta potential of 11.8 ± 0.68 mV. And the ultrasonic imaging indicated that the PDLIM5 siRNA NBs maintain the same signals as the microbubbles (SonoVue). Under the optimized conditions of 0.5 W/m^2^ ultrasound intensity and 1 min irradiation duration, the highest transfection efficiency of PC9GR cells was 90.23 ± 1.45%, which resulted in the inhibition of PDLIM5 mRNA and protein expression. More importantly, the anti-tumor effect of fabricated PDLIM5siRNA-NBs with the help of ultrasound irradiation has been demonstrated to significantly inhibit tumor cell growth and promote apoptosis. Therefore, NBs carrying PDLIM5siRNA may have the potential to act as gene vectors combined with ultrasound irradiation to antagonize drug resistance for NSCLC.

## 1. Introduction

Lung cancer is the main reason for cancer deaths worldwide. According to CA Cancer J Clinic's 2018 Cancer Statistics, lung cancer continues to occupy the first place in the death toll of malignant tumors in the United States, and the estimated annual death toll of malignant tumors accounts for more than 1/4 of the total (Siegel et al., 2018). The WHO points out that lung cancer can be divided into small cell lung cancer (SCLC) and non-small cell lung cancer (NSCLC), of which NSCLC accounts for 85% (Oser et al., 2015). Along with surgery and radiation, chemotherapy is one of the most common treatments for lung cancer. Although the standard platinum chemotherapy remains the cornerstone of systemic cancer therapy for those with advanced lung cancer (Planchard et al., 2014), the discovery of lung cancer-driving mutation genes in recent decades has greatly changed the treatment of NSCLC. Gene-targeted therapy has been developed as a promising therapeutic method. As one of the molecular therapies, epidermal growth factor tyrosine kinase inhibitors (EGFR-TKIS) have been approved by the American Society of Clinical Oncology (ASCO) and the National Comprehensive Cancer Network (NCCN) as the therapy of choice for patients with EGFR mutations. EGFR-TKIs, as competitive inhibitors of adenosine triphosphate (ATP) in the TKIS domain of EGFR, inhibit abnormal proliferation and differentiation of tumor cells by inhibiting EGFR and blocking its downstream signal transduction, and ultimately inhibiting tumor growth and progression. However, there was no significant improvement in overall survival (Tripathi et al., 2017), mainly because NSCLC is prone to drug resistance during treatment. The average progression-free survival (PFS) is only about 1 year (Wu & Shih, 2018). Therefore, how to overcome drug resistance is still challenging.

It has been found PDLIM5 gene is overexpressed in NSCLC and was associated with a poor prognosis of NSCLC. Studies have shown that PDLIM5 is a member of the PDZ-LIM protein family, also known as Enigma Homolog (ENH). It is often used as a scaffold protein to be involved in the signal-transducing regulation of membrane-associated proteins, cytoskeletal proteins, various signaling molecules, as well as the progression of various tumors (Edlund et al., 2012; Wang et al., 2016). Recent studies have shown that PDLIM5 regulation of NSCLC drug resistance is related to AMPK/TSC2/mTORC1 signaling pathway. The possible mechanisms are as follows: (1) PDLIM5, as a substrate of adenosine activated protein kinase AMPK, is a major signaling molecule to regulate cell proliferation under the AMPK activation environment. PDLIM5 can directly bind to AMPK, maintain AMPK activation, inhibit AMPK degradation. By knocking out a tumor or intracellular PDLIM5, it can inhibit the proliferation of tumor cells, induce cell cycle stagnation, and promote cell apoptosis (Li et al., 2015; Liu et al., 2017). Activation of AMPK can decrease the metabolic activity of tumor cells under metabolic stress, such as sugar starvation, thereby enhancing the viability of tumor cells. (2) In the case of increased AMPK activity, PDLIM5 is a major signaling molecule to regulate cell migration. The activated AMPK can directly phosphorylate SER177 of PDLIM5 attached to the actin filaments, thus causing changes in the actin skeleton structure. For example, the formation of defective patchy pseudopodia, ventral stress fiber enhancement, and adhesion spots to the edge of cells, thus inhibiting cell migration and invasion (Yan et al., 2015). (3) Activation of AMPK further inhibits its downstream substrate mTOR through the TSC1/2 complex, thus enabling cell survival and proliferation, helping cells adapt to a stressful environment, promoting cell survival, and leading to tumor drug resistance (Kim et al., 2018). Therefore, the specific intervention of PDLIM5 expression may relieve the inhibitory effect of AMPK activation on mTOR under stress state, and then affect cell proliferation, accelerate cell apoptosis, and improve drug sensitivity of tumor cells to EGFR-TKIS. Therefore, the enhanced expression level of PDLIM5 is expected to be a new method for the treatment of drug-resistant NSCLC.

Including RNA interference (RNAi), gene therapy in cancer treatment has been broadly applied due to the new gene carriers. However, the lack of a non-toxic, reliable, and efficient RNAi delivery system has hindered the progress and clinical use of gene therapy (Oba et al., 2008). Lentiviral vectors indeed provide efficient gene delivery; however, subsequent immunotoxicity and lack of site-specificity make it difficult to achieve a stable and persistent state of gene expression. It is much safer for non-viral vectors in experiments, although they are limited by low transfection efficiency and critical technical challenges (Jing et al., 2015). In recent years, a new technique of carrying genes/drugs to treat diseases by ultrasound microbubbles (MBs)/nanobubbles (NBs) contrast agent has attracted great attention (Wang et al., 2014). NBs, submicron bubbles, typically 150–500 nm in diameter (Bosca et al., 2018), are an attractive vector for therapeutic drug/gene delivery and have been shown to improve cancer aggregation and retention compared to MBs (Nittayacharn et al., 2019; Wu et al., 2019). The gene location-controlled release mediated by ultrasound-mediated nanobubble destruction (UMND) maintains a variety of advantages: (1) The cavitation effect or mechanical effect of nanobubbles can make it easier for the carried genes/drugs to reach the tissue cells through the ruptured microvessel and endothelial cell space (Deng et al., 2014); (2) It can better protect the carrying gene/drug, reduce the loss of gene/drug before reaching the lesion, improve the targeting, increase the local release concentration, enhance the curative effect, and reduce the toxic and side effects on normal tissues (Wu et al., 2018); (3) The effects of micro acoustic flow, shock wave and fluid microjet generated under ultrasonic irradiation can further improve the targeted transfection or drug delivery of carrying genes/drugs (Zhao et al., 2018); In addition, in our previous studies, we have successfully used traditional ultrasound microbubble contrast agents as gene vectors to target tumor microvessels, the results showed that it restrained tumors growth and promoted apoptosis in tumor cells (Nie et al., 2006; Nie et al., 2008). Based on the previous study, this study aims to design novel NBs, study their response to ultrasound irradiation and transfection efficiency, and confirm the hypothesis that PDLIM5 siRNA combined with ultrasound irradiation can downregulate PDLIM5 expression in NSCLC and inhibit tumor growth *in vitro* by the mechanism of EGFR-TKIS resistance.

## 2. Material and methods

### 2.1. Material and chemicals

1,2-distearoyl-sn-glycerol-3-phosphoethanolamine-N- [methoxy (polyethylene glycol)-2000] (DSPE-PEG2000, purity > 99%), 1,2-distearoyl-sn-glycerol-3-phosphocholine (DSPC), and 1,2-dioleoacyl-3-trimethylammonium-propane (DOTAP) were obtained from Shanghai Ponsure Biotech Inc. (Shanghai, China). Gas sulfuric hexafluoride (SF-6) was obtained from Qiangyuan Gas Co., Ltd. (Wuhu, China). Cell Counting Kit-8 (CCK-8) was purchased from AbmMole Bioscience, Ltd. (Shanghai, China). Fetal bovine serum (FBS), cell culture medium RPMI 1640, trypsin, and Opti-MEM (1×) were obtained from GIBCO BRL (Grand Island, USA). SonoVue (Bracco, Switzerland) is the second generation of commercial micron-grade contrast agents, which consists of phospholipase-coated SF_-6_ microbubbles as control (Schneider, 1999). 4,6-diamino-2-phenyl indoles (DAPI) was obtained from Sigma-Aldrich (St. Louis, MO, USA). Gefitinib was obtained from Selleck Corp. (Houston, TX, USA).

Three types of siRNA duplexes were designed to target the PDLIM5 siRNA gene, and the FITC-labeled scrambled siRNA (FITC-SCR siRNA) was obtained from Santa Cruz Biotechnology (CA, USA). The sequences of siRNA were shown in Table 1. PDLIM5 siRNA consists of three targeted 19–25 base pairs of siRNAs devised to knock down PDLIM5 gene expression. gene expression. The configuration containing PDLIM-a, PDLIM-b, and PDLIM-c (equal molar ratio) was named PDLIM5-siRNA.

**Table 1.** Sequences of PDLIM5 siRNA and negative control.

Gene		Sense	Antisense
PDLIM5	PDLIM5-a	5′-CAUGACACUUGCUUUGUAtt-3′	5′-UACAAAGCAAGUGUCAUGCttt-3′
	PDLIM5-b	5′-CAUGAACCUCCUAAGUUAttt-3′	5′-UAACUUAGGAGGUUCAUGGttt-3′
	PDLIM5-c	5′-AAGGCAUCUGUCCUGAAAttt-3′	5′-UUUCAGGACAGAUGCCUUGttt-3′
FITC-Scrambled siRNA (for a negative control)	FITC-SCR	5′-UCUCCGAACGUGUCACGUttt-3′	5′-ACGUGACACGUUCGGAGAAttt-3′

### 2.2. Cell culture

The cell line PC9 cells (NSCLC) and H16BE were kindly provided by the department of pathology, School of Basic Medicine, Guangzhou Medical University, Guangzhou, China. For the PC9GR cells, PC9 cells were firstly cultured in a culture medium with 2 μmol/L gefitinib to gradually establish their drug resistance, then the gefitinib 4 μmol/L concentration increased to 60 μmol/L in the culture medium when subculture. These cells that stably grew and proliferated in the culture medium with a gefitinib concentration of 2 μmol/L could be used in the following experiments. All cells were cultured in a mixture of 90% RPMI 1640 medium and 10% FBS in a humidified incubator at 37 °C, 5% CO_2_.

### 2.3. Fabrication of NBs and siRNA-NBs

The NBs were prepared according to the reported method in collaboration with Southeast University (Jin et al., 2020). Briefly, a lipid mixture of 0.1 mg/mL DSPC and 0.1 mg/mL DSPE-PEG2000 was added to a hydration solution consisting of glycerol (V/V, 10%), normal saline (V/V, 90%), and ethanol (4 wt.%) for ultrasonic dispersion for 5 min. Next, after the phospholipid materials were evenly mixed, 0.1 mg/mL DOTAP was added and quickly heated to 70 °C to drain the dissolved oxygen, carbon dioxide, and other gases in the solution for at least 5 min. At room temperature, a saturated buffer containing sulfuric hexafluoride gas was added to a closed container, and at least three times the volume of SF_6_ gas was used to remove the air in the container. The phospholipid material was preheated to 70 °C to uniformly disperse the phospholipid material to prevent impurities, such as liposomes and phospholipid micelles from affecting the bubble preparation. The bubbles were quickly heated to 60 °C and then cooled to 20 °C after 0.5 mm diameter micropores and high shear force. Lastly, Stable cationic lipid bubbles were obtained. 1.2 nmol (0.6 µM) siRNA was mixed with 2 mL of NBs. The temperature was rapidly heated to 70 °C for 10 s, and then the temperature was rapidly cooled to 60 °C. siRNA and cationic bubbles passed through the micropores within 1 min, and then cooled to 4 °C quickly, thus obtaining NBs carrying siRNA on the surface.

### 2.4. Observation of the size, distribution, and morphology of blank NBs and prepared siRNA-NBs

#### 2.4.1. Measurement of particles size distribution

The size and potential of both the blank NBs and siRNA-NBs were measured with a Malvern Nano analyzer (Zeta-Sizer, Malvern Instrument, UK). The concentration of NBs was detected using Coulter Multisizer Particle Counter (Multi4e, Beckman, USA). The NBs have diluted adequately with double-distilled water. Thus, NBs’ average size, surface potential, and concentration were obtained.

#### 2.4.2. Morphological observations siRNA-NBs under TEM

Morphological observations of siRNA-NBs were operated under transmission electron microscopy (TEM, HT-7800; HITACHI, Tokyo, Japan). For sample preparation, a 10uL sample of the solution was uniformly spread on a 400-mesh carbon-coated copper net, dried at room temperature (RT), and then observed by TEM.

### 2.5. Ultrasound image in vitro

*In vitro*, acoustic imaging of NBs was performed using self-made agar, which was composed of 5% agar, 10% glycerin, and 85% double distilled water. A 1.5 mL centrifuge tube was inserted into the gel, and the gel was solidified to prepare the sample adding hold. Ultrasound image was performed using a microimaging system (Visual Sonics Vevo 2100, USA). The imaging of the ultrasound image system was set to a frequency of 21 MHz, an ultrasound intensity of 4%, and a gain of 30 dB. In all ultrasound imaging acquisitions, the parameters did not change. To determine whether NBs would have the characteristics of ultrasonic contrast imaging, PBS, freshly prepared NBs, and SonoVue microbubbles dispersions were injected into the gel mold for imaging, respectively. In addition, to observe the blasting of nanobubbles and microbubbles, the nanobubbles and microbubbles were irradiated with ultrasonic irradiation, respectively (ultrasound intensity of 1 MPa, the duty cycle of 50%, duration of 8 min). Analysis of the average power intensity was conducted in the region of interest (ROI).

### 2.6. Determination of connection of NBs and FITC-SCR siRNA

The condition of NBs carrying siRNA was confirmed by Laser Scanning Confocal Microscope(LSCM). To evaluate the binding of siRNA to NBs, a FITC-labeled SCR siRNA was used to replace the PDLIM5 siRNA. Ten microliters upper suspension sample was observed to drop uniformly on the slide and the condition of NBs binding siRNA was also observed by LSCM (Zeiss LSM 880 confocal workstation with Airyscan, Germany). The fluorescence distribution on the NBs surface was detected to evaluate whether FITC-SCR siRNA could effectively bind NBs. In addition, the PDLIM5 siRNA-NBs were newly prepared and centrifuged at 600 rpm for 5 min, then stored at 4 °C for 30 min. The upper layer of the suspension contained PDLIM5 siRNA bound to NBs, while the lower layer contained unbound and free PDLIM5 siRNA. Then, the concentration of unbound PDLIM5 siRNA was measured in the lower suspension by a spectrophotometer (NanoDrop2000/2000C, Thermo Scientific, USA), and the amount of unbound PDLIM5 siRNA was calculated (Wu et al., 2018). The experiment was repeated three times. Encapsulation efficiency of NBs (EE, %) = (total amount of siRNA in NBs suspension − the total amount of unbound siRNA)/total amount of siRNA in NBs suspension.

### 2.7. Cytotoxicity assay of NBs-siRNA in vitro

PC9GR cells were uniformly planted in 96-well plates with a density of 5000 cells per well, given 100 µL RPMI-1640 medium containing 10% fetal bovine serum, and then cultured at 37 °C with 5% CO2 overnight. The cytotoxicity assays were divided into two groups: group I, the original medium was changed to a fresh medium containing PBS (Control), blank NBs, SCR siRNA-NBs or Naked SCR siRNA (at siRNA-NBs volume 10 µL/well or siRNA 6 pmol/well) and cultured with PC9GR cells for 12 h. PC9GR was replaced with fresh medium and cultured for 48 h; group II, the original medium was changed to a fresh medium containing PBS (Control), different concentrations of blank NBs, or SCR siRNA-NBs (at 1.0 × 10^6^, 5.0 × 10^6^, 1.0 × 10^7^, 5.0 × 10^7^, 1.0 × 10^8^, and 5.0 × 10^8^ bubbles/mL) and cultured with PC9GR cells for 12 h. After the cells were rinsed with PBS twice, 100 µL of new culture medium was replaced in each well, and 10 µL Cell Counting Kit-8 (CCK-8) was added, then the cells were put back into the incubator for further incubation for 3 h. Finally, the absorbance was measured at 450 nm with an enzyme plate meter (Bio Tek, Proton Instrument Co., Ltd., USA). The above experiment process is repeated three times.

### 2.8. PC9GR cells transfection

For the transfection efficiency assay, PC9GR cells were plated in 6-well plates with a density of 2 × 10^5^ cells per well and cultured overnight in 2 mL of RPMI-1640 medium attaching 10% FBS. The original medium was changed to a fresh medium containing 0.2 mL of siRNA-NBs (120 pmol siRNA) in each well and then cultured at cells incubator for 5 min, followed by ultrasonic irradiation. Ultrasonic irradiation was performed with a therapeutic US system (Model UGT 1025, Institute of ultrasound imaging, Chongqing medical university, Sichuan, China). Ultrasonic irradiation fixed conditions of the instrument are set as follows: the operating frequency of the transducer is 1.0–1.1 MHz, the duty cycle is 50%, and the pulse repetition frequency is 1 kHz. After the coupling agent was covered on the ultrasonic probe, the probe was wrapped with a sterilized material membrane and placed on the top of the plate, deep below the surface of the medium, without touching the adherent cells. After that, they were incubated for 12 h and replaced with a 2 mL fresh medium for subsequent experiments.

### 2.9. Evaluation of cells transfection efficiency in transfection of NBs loading FITC-SCR

To investigate the optimal transfection efficiency of ultrasound irradiation to optimize the conditions of gene transfection, ultrasonic irradiation was conducted with variations in several parameters, including ultrasound intensity and the irradiation duration. Firstly, in terms of ultrasound intensity, PC9GR cells were divided into four groups, namely the control group (the cells without any intervention), 0.26, 0.5, and 1.03 W/m^2^, and the irradiation time was 1 min to determine the influence of ultrasound intensity. Additional four groups were performed to determine the effect of irradiation duration, which was the control group (the cells without any intervention), 30 s, 1 min, and 3 min under the ultrasound intensity of 0.5 W/m^2^. After ultrasound intervention, the cells were incubated for 12 h, replaced with fresh medium, and continued to be cultured for 24 h. Cell transfection efficiency was evaluated using confocal laser scanning microscopy (CLSM) and Flow cytometry analysis. To prepare confocal laser samples, the cells were first rinsed with PBS twice and fixed with 4% formaldehyde for 10 min, then stained with DAPI (Beyotime Biotech, China) for 15 min. FITC and DAPI were activated at 492 and 359 nm, and were emitted at 525 and 460 nm, respectively. In addition, detection of FITC-SCR content within the cell was performed using flow cytometry (CytoFLEX, Beckman Coulter, USA). Forty-eight hours after transfection, the cells were digested with trypsin and collected, rinsed with PBS twice, and then re-suspended with 500 µL PBS. Normal cells without any intervention were used as controls. The above experiment process is repeated three times.

### 2.10. Cell viability detection

In the cell viability experiment, seven groups were established with different treatments and conditions operated (Table 2). PC9GR cells were uniformly planted in 96-well plates with a density of 5000 cells per well, given 100 µL RPMI-1640 medium containing 10% fetal bovine serum, and then cultured at 37 °C with 5% CO_2_ overnight. The appropriate ultrasound conditions were used: ultrasound frequency 1.0–1.1 MHz, ultrasound intensity of 0.5 W/m^2^, irradiation duration of 1 min. All groups were treated with 2 μmol/L gefitinib at 24 h after transfection. Cell viability was measured from day 1 to day 6 after transfection by CCK-8 assay. Each group was divided into 5 equivalent well tests and 5 negative control Wells. The absorbance at 450 nm [*D*_450 nm_] was tested with a microplate reader. The absorbance at 450 nm was measured with a microplate reader. Taking time as X-axis and absorbance as Y-axis, the cell growth curve was acquired, and the cell growth inhibition rate (IR) was obtained by formula (1):

(1) $$\mathrm{IR}\;(\%)\;=\;[1-(\text{Mean of }D_{450\;\mathrm{nm}}\text{ of siRNA transfection groups }-\text{ Mean of }D_{450\;\mathrm{nm}}\text{ of blank groups})/(\text{Mean of }D_{450\;\mathrm{nm}}\text{ of negative control groups}-\text{Mean of }D_{450\;\mathrm{nm}}\text{ of Blank})]\times 100\%$$

**Table 2.** Groups of experiments and corresponding treatment.

Groups	Intervening measure	Abbreviation
1	Blank	Blank
2	Nonsense siRNA	SCR
3	Nanobubbles carrying nonsense siRNA	SCR-NB
4	Bare PDLIM5 siRNA	PDLIM5siRNA
5	Bare PDLIM5 siRNA + ultrasonic irradiation	PDLIM5siRNA + US
6	Nanobubbles carrying PDLIM5 siRNA	PDLIM5siRNA-NB
7	Nanobubbles carrying PDLIM5 siRNA + ultrasonic irradiation	PDLIM5siRNA-NB + US

*Note*. All groups were treated with 2 μmol/L gefitinib at 24 h after transfection.

### 2.11. Apoptotic changes in PC9GR cells

The Cell Apoptosis Assay Kit was purchased from BD Biosciences, Inc. (CA, USA) and applied according to instructions. The experiment was divided into seven groups as shown in Table 2. PC9GR cells were collected at 48 h after transfection and re-suspended with binding buffer at a density of 1 × 10^6^ cells/ml, then incubated with 5 μL Annexin V–FITC and 5 μL PI (20 μg/ml) for 15 min in darkness at RT. A 400 μL binding buffer was added to the cells and was immediately detected at 630 and 525 nm by flow cytometry. Data were analyzed by Flow Jo 7.65 software.

### 2.12. Molecular biological detection

#### 2.12.1. mRNA level of PDLIM5, Bcl2, Bax, and Caspase3 genes by RT-PCR

In the experiment, seven groups were also established with different treatments and conditions operated (Table 2). PC9GR cells were cultured and seeded in 6-well plates at 1 × 10^5^ cells/mL density overnight. Under appropriate ultrasound conditions: ultrasound frequency 1.0–1.1 MHz, ultrasound intensity of 0.5 W, irradiation duration of 1 min. All groups were treated with 2 μmol/L gefitinib at 24 h after transfection. The PC9GR cells were cultured for 48 h after ultrasonic irradiation, and their total RNA was obtained by Trizol Reagent Kit (Invitrogen, Germany) according to the instructions. RT-PCR was performed by Roche Light Cycle 96, Germany. The total RNA was conducted gDNA and reverse transcription using Prime Script RT reagent Kit (Accurate Biotechnology, Hunan, China) and the mRNA expression of GAPDH was detected as an internal reference. Amplified primer sequences were synthesized (S1). RT-PCR was conducted in a mixture of 1 μL primers, 8 μL SYBR Green Master (ROX) reagent (Accurate Biotechnology, Hunan, China), 2 μL cDNA sample and H_2_O up to 20 μL. gDNA procedure was 42 °C for 5 min, 4 °C keep. The reverse transcription parameters were incubated at 37 °C for 15 min, 85 °C for 5 s, and 4 °C keep. The conditions of amplification were 95 °C for 30 s; 40 cycles of 95 °C for 5 s, 60 °C for 30 s; 95 °C for 5 s, 60 °C for 60 s, 95 °C, 1 s; 50 °C for 30 s.

#### 2.12.2. Protein expressions of PDLIM5, Bcl2, Bax, and Caspase3 genes by Western blot analysis

The abovementioned transfection technique with the appropriate ultrasound conditions was used to transfect. All groups were treated with 2 μmol/L gefitinib at 24 h after transfection. The PC9GR cells were cultured for 72 h after ultrasonic irradiation, and their total proteins were obtained from the cells by protein extraction kit (Merk, Germany) and BCA assay kit (Labgic Technology, Beijing, China) separately. The protein concentration was determined by a microplate reader. According to the molecular weight of the protein, select the appropriate separation glue and concentration glue, 20 mg total protein were separated, moved to PVDF membranes, blocked in 5% skim milk, and at RT on a shaking table for 30 min with rabbit antibody against PDLIM5 (1:1000 dilution; Proteintech, Wuhan, China), rabbit antibody against Bcl2, rabbit antibody against Bax, rabbit antibody against cleaved caspase-3 (1:1000 dilution; Cell Signaling Technology, Danvers, USA), and rabbit antibody against GAPDH (1:10,000 dilution; Cell Signaling Technology, Danvers, USA) as an internal reference. Protein signals were measured by chemiluminescence introduction (Tanon 5200 Multi, Shanghai, China).

The samples were normalized by GAPDH. The inhibition rate of the target gene protein expression was calculated by equation (2): inhibition rate = [1−(density of treatment group PDLIM5/density of treatment group GAPDH)/(density of control group PDLIM5/density of control group GAPDH)] × 100%. (2) The above experiment process is repeated three times.

### 2.13. Statistical analysis

The Data were expressed as the mean ± standard deviation (SD) and statistically assayed by GraphPad Prism Software version 8.0.2 (CA, USA). Statistical significances were performed using Student’s *t*-test between different groups and a one-way ANOVA followed by Bonferroni tests. If *p*-values of <.05, the difference was considered significant. All experiments were repeated at least three times.

## 3. Results and discussion

### 3.1. Determination of distribution, the size, and morphologic observation of blank NBs and prepared NBs with siRNA

**Figure 1:**
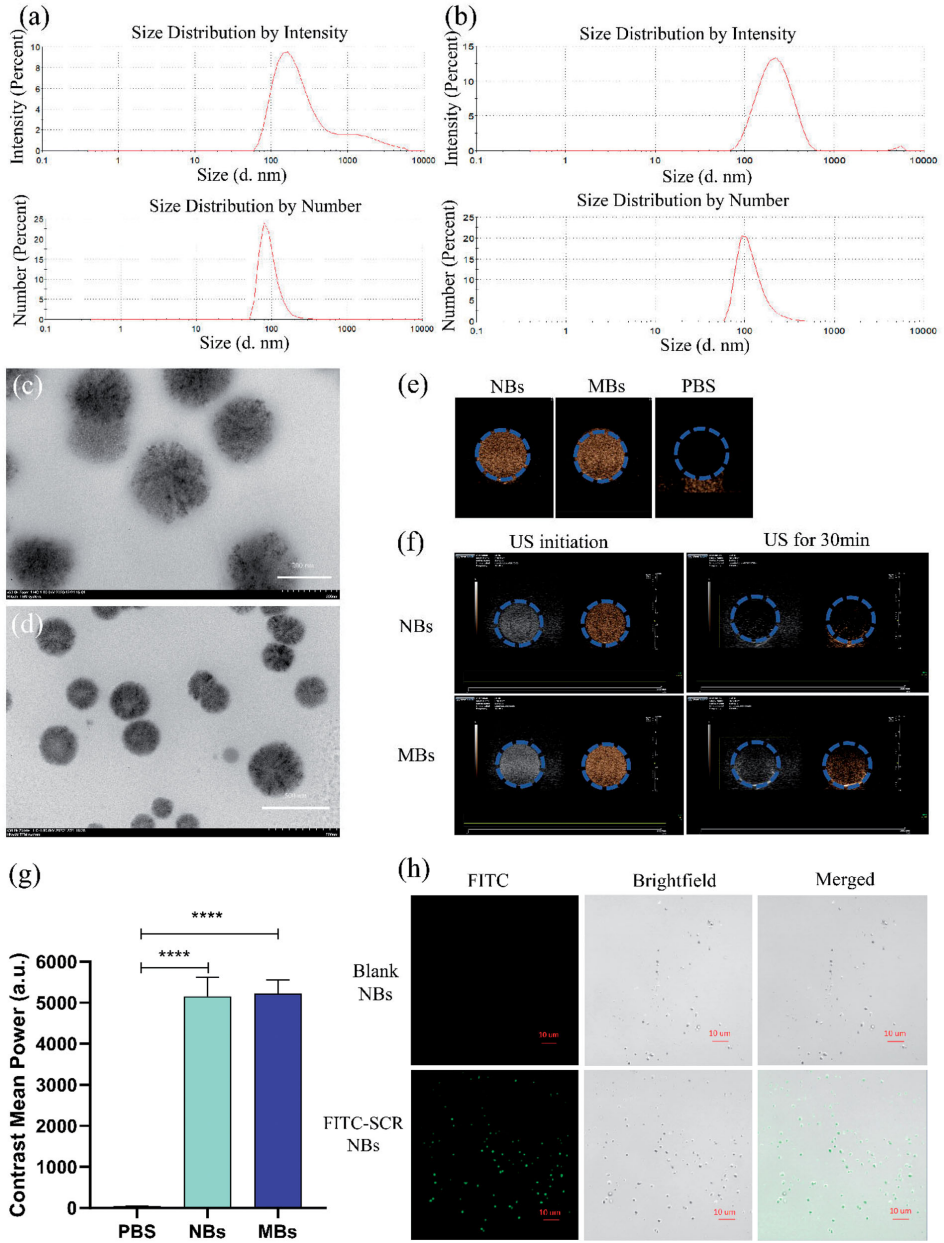
Characterization of NBs binding with FITC-SCR siRNA. The size and zeta potential of NBs (a) and PDLIM5si RNA-NBs complex (b). TEM observation of the shape and size of NBs-siRNA, magnified by 60,000 times (c) and 30,000 times (d). *In vitro* ultrasound images of NBs. (e) Ultrasound images of NBs (left), MBs (middle), and PBS (right). (f) The ultrasonic contrast intensity of NBs and MBs at the beginning and 30 min after ultrasound irradiation. (g) Quantitative analysis of data obtained from e. (h) NBs with FITC-labeled siRNA was observed under CLSM. The blank NBs showed no fluorescence, while FITC-SCR-NBs showed significant fluorescence. The green fluorescence was FITC-SCR (×1,000).

The size distribution of the blank NBs, and prepared NBs with siRNA is shown in Figures 1(a,b). The average diameter of blank NBs was 191.6 ± 0.50 nm, and the Zeta potential was 11.8 ± 0.68 mV. PDLIM5si RNA -NBs complex mean diameter was 199.2 ± 1.09 nm and Zeta potential was −2.2 ± 0.13 mV. The prepared NBs concentration was 1.4 ± 0.11 × 10^9^/mL, which was higher than that of SonoVue microbubbles (9.0 ± 0.61 × 10^8^/mL). The size and distribution of siRNA-NBs were uniforms by TEM (Figures 1(c,d)). The prepared NBs are spherical and uniform in size, and the size of NBs is about 200 nm, which is consistent with the Malvern Nano analyzer's results for NBs size.

### 3.2. Ultrasound image in vitro

The properties of NBs as ultrasound contrast agents were evaluated by measuring and comparing the intensity of Contrast-enhanced ultrasonic (CEUS) between the NBs and PBS (as control). The contrast images of NBs and MBs showed significant visual differences compared with PBS, while there was no significant difference between NBs and MBs (Figure 1(e)). The results were verified by quantitative analysis (Figure 1(g)). NBs and MBs can be enhanced by echogenic ultrasound images, indicating that the experimental materials are bubbles with good echogenic characteristics. After continuous ultrasound irradiation for 30 min, the ultrasonic signal strength was fully reduced due to the collapse of bubbles (Figure 1(f)). This proved that NBs were destroyed by ultrasound irradiation, and the intensity of CEUS was decreased.

### 3.3. Observation of the condition of NBs carrying siRNA

The strategy of combining siRNA with NBs was confirmed by CLSM to be effective; Only nanobubbles bound to FITC-labeled SCR indicated green fluorescence, and no fluorescence was shown for blank NBs (Figure 1(h)). The encapsulation rate of PDLIM5siRNA carried by NBs is 93 ± 2.6%, indicating that a good connection between PDLIM5siRNA and NBs can be achieved through the principle of positive and negative charge attraction.

### 3.4. Cytotoxicity assay of NBs-siRNA in vitro

**Figure 2.**
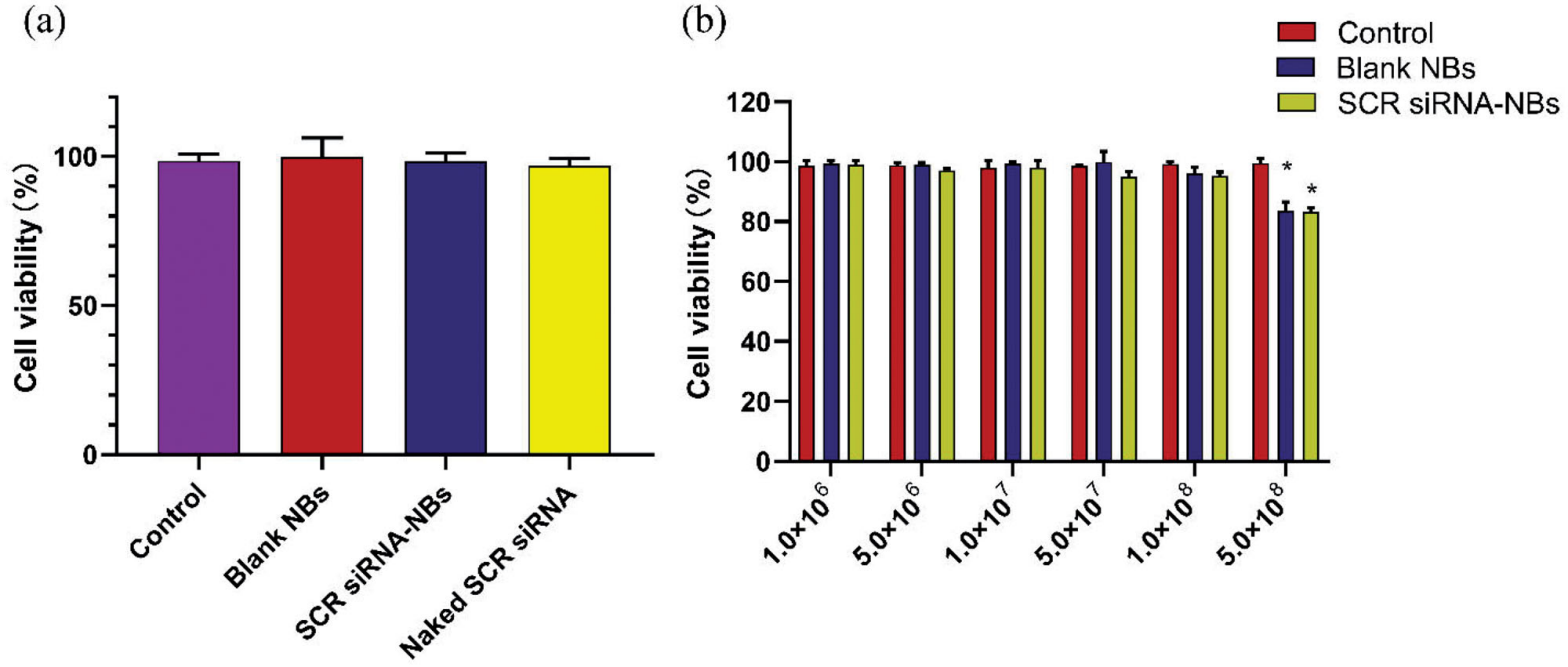
*In vitro* biocompatibility of NBs was tested by CCK-8 assay. (a) The original medium was changed to a fresh medium containing PBS (Control), blank NBs, or SCR siRNA-NBs (at siRNA-NBs volume 10 µL/well or siRNA 6 pmol/well). (b) The original medium was changed to a fresh medium containing PBS (Control), different concentrations of blank NBs, or SCR siRNA-NBs (at 1.0 × 10^6^, 5.0 × 10^6^, 1.0 × 10^7^, 5.0 × 10^7^, 1.0 × 10^8^, and 5.0 × 10^8^ bubbles/mL). *p < .05, compared with Control.

The cytotoxicity of increasing concentration NBs to PC9GR cells was detected by CCK-8 assay. As shown in Figure 2(a), blank NBs and SCR siRNA-NBs exhibited had no significant toxicity, and the viability of the cells was over 95%. Compared with the control group, there was no significant difference in cell viability when the concentration of NBs ranged from 1.0 × 10^6^ to 1.0 × 10^8^/mL (*p* > .05). When the concentration of NBs was increased to 5.0 × 10^8^/mL, the cell viability was decreased significantly (*p* < .05), but still more than 80% (Figure 2(b)). siRNA-NBs exhibited dose-dependent cytotoxicity.

### 3.5. Difference in the expression level of PDLIM5 gene in H16BE, PC9, and PC9GR cell

**Figure 3.**
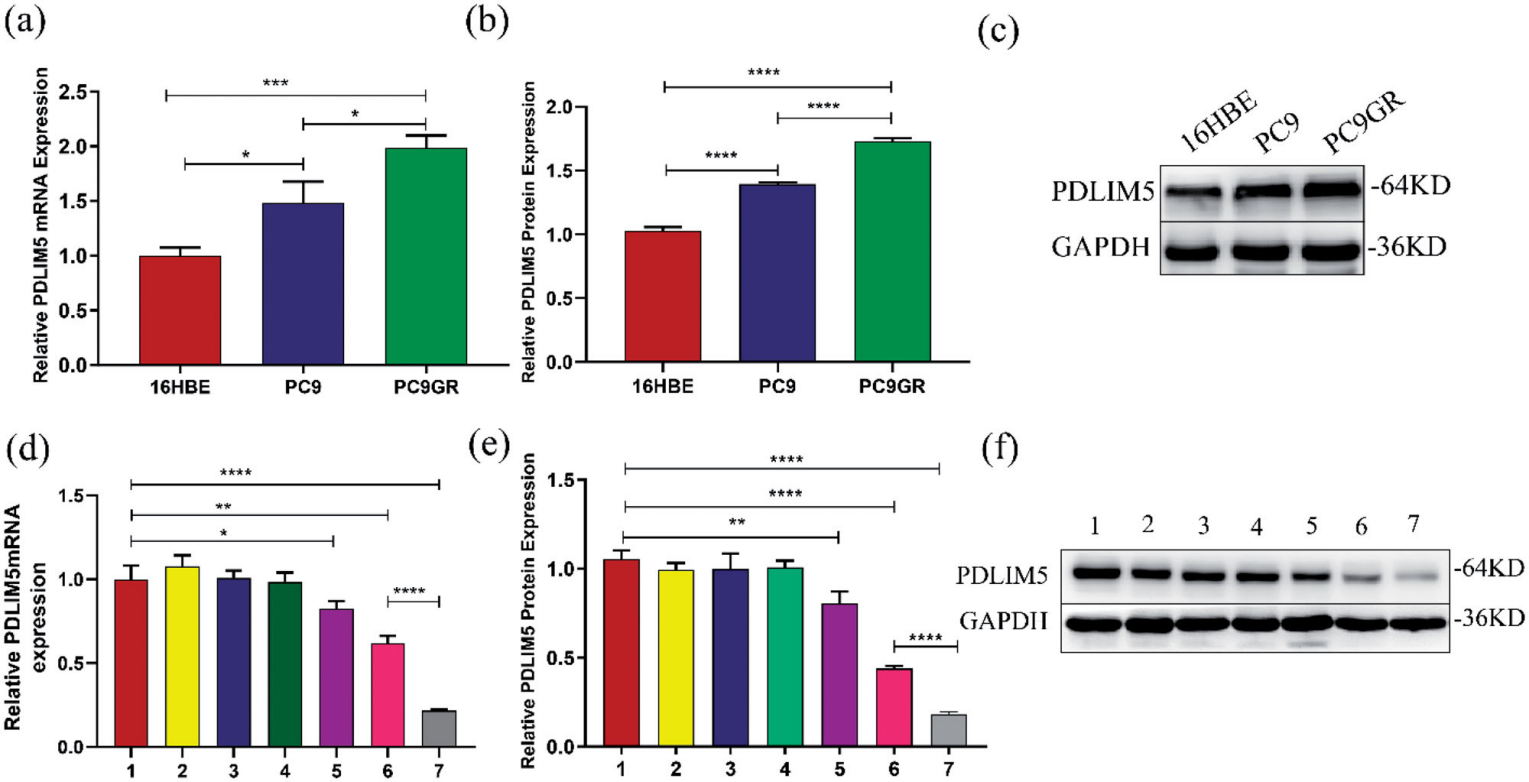
Expression differences of PDLIM5 in PC9GR cells. The mRNA and protein expression differences of PDLIM5 in H16BE, PC9, and PC9GR cells were detected using RT-PCR (a) and Western blot (b,c). The knockdown of the PDLIM5 gene in PC9GR cells was detected: the expression of PDLIM5 mRNA in PC9GR cells was detected in seven groups by RT-PCR (d). The expression of PDLIM5 protein in PC9GR cells was tested in seven groups by Western blot (e,f). GAPDH acted as an internal reference. Groups 1–7 represented as Table 2: (Group 1) Blank, (Group 2) SCR, (Group 3) SCR-NB, (Group 4) PDLIM5siRNA, (Group 5) PDLIM5siRNA + US, (Group 6) PDLIM5siRNA-NB, and (Group 7) PDLIM5siRNA-NB + US. *p < .05, **p < .01, ***p < .005, ****p <.001, Comparison between the two groups.

The expression of the PDLIM5 gene in 16HBE, PC9, and PC9GR cells was detected using QT-PCR and Western blot. The result showed that compared with 16HBE, the mRNA and protein contents of PDLIM5 increased in PC9 and PC9GR, especially in PC9GR (Figures 3(a–c)). These findings suggest that PDLIM5 is overexpressed in NSCLC drug-resistant PC9GR cell lines.

### 3.6. Transfection efficiency assay in PC9GR cells

**Figure 4.**
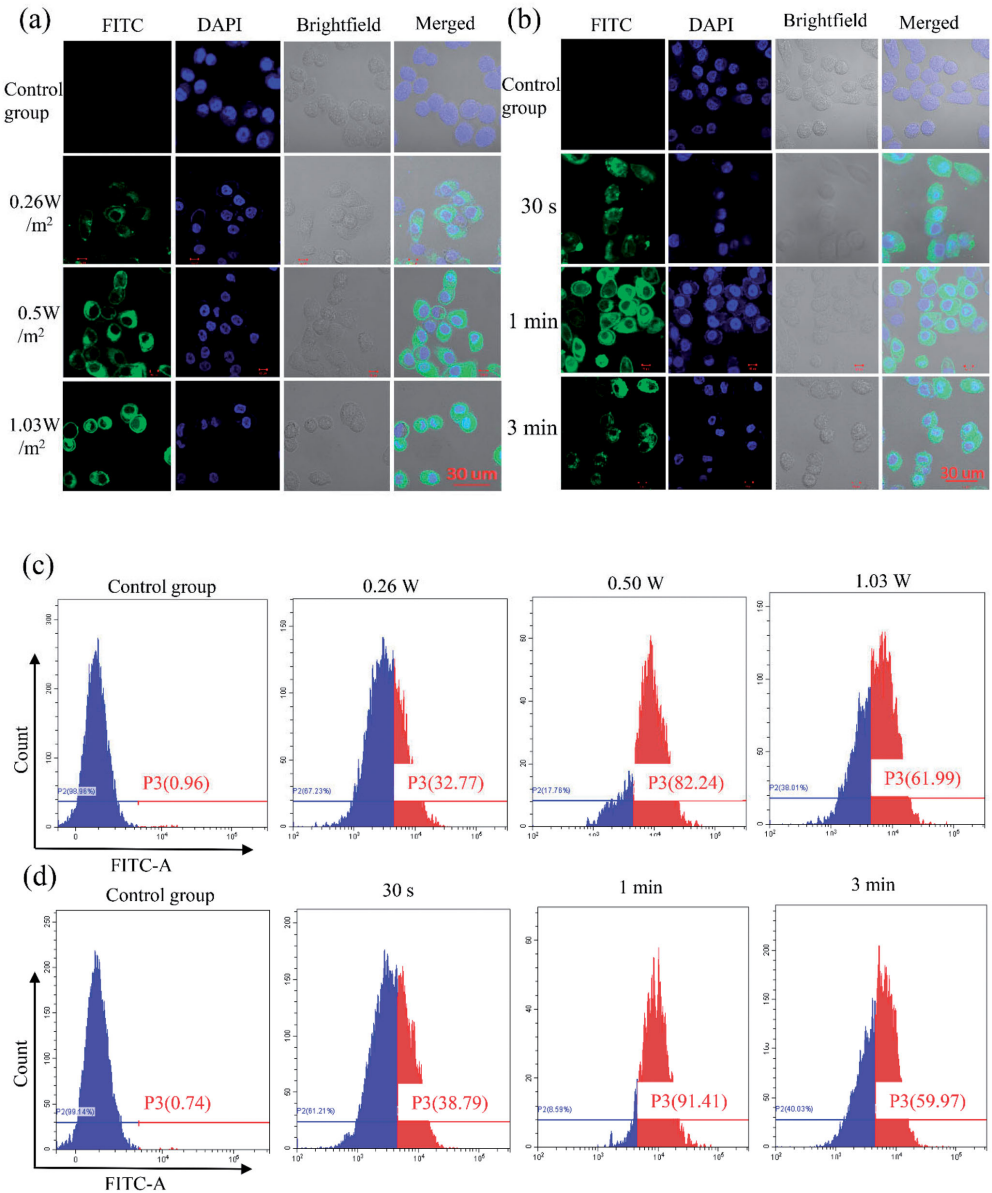
*In vitro* gene transfection efficiency was evaluated for PC9GR cells. (a,b) Images of PC9GR cells transfected with FITC-SCR siRNA detected by LSCM. Four groups were performed with different ultrasound intensity, namely the control group (the cells without any intervention), 0.26, 0.5, and 1.03 W/m^2^, and the irradiation time was 1 min (a). Additional four groups were performed to evaluate the effect of the duration, which was the control group (the cells without any intervention), 30 s, 1 min, and 3 min under the ultrasound intensity of 0.5 W/m^2^ (b). The cytoplasm of 9GR cells in the experimental group showed green fluorescence, but it was not detected in the nucleus. Blue (DAPI) represented the nucleus, while green (FITC) implied FITC-labeled siRNA. (c,d) Flow cytometry was used to detect the proportion of transfected cells in PC9GR cells under different ultrasound irradiation parameters. Four groups were performed with different ultrasound intensity, namely divided into four groups, namely, control group (the cells without any intervention), 0.26, 0.5, and 1.03 W/m^2^, and the irradiation time was 1 min (c). Additional four groups were performed to determine the effect of irradiation duration, which was the control group (the cells without any intervention), 30 s, 1 min, and 3 min under the ultrasound intensity of 0.5 W/m^2^ (d). (P3: The proportion of green fluorescent cells successfully transfected with FITC-labeled siRNA was detected by Flow cytometry).

Normal tissue microvascular endothelial gap density, the endothelial cell structure is complete, and solid tumor endothelial new pore between 380 and 780 nm, endothelial cell structural integrity is poorer, therefore, enhanced permeability and retention (EPR) effect, is considered a complete mechanism of targeted therapy of tumor tissue passive (Torchilin, 2011; Duan et al., 2020). Compared with other nanoparticles, NBs have the special property of ‘ultrasonic cavitation,’ which is caused by ultrasound irradiation, forming a gap of about 300 nm in diameter on the cell membrane and increasing the permeability of cells (Hancock et al., 2009). To assay transfection efficiency of cell targeting effects which ultrasound irradiation and siRNA-NBs, firstly, green fluorescence (indicative of FITC-labeled siRNA) was detected at 24 h after transfection by CLSM. As shown in Figure 4, it was obvious that no green fluorescence was detected in the control group, while the cytoplasm of PC9GR cells in the experimental group showed green fluorescence, and it was not detected in the nucleus. especially, the image of the group which the intensity of 0.5 W/m^2^ and the impact of the duration of 1 min showed visible numerous cells carrying green fluorescence (Figures 4(a,b)). Notably, acoustic pressure over 1 W/m^2^ and the impact of the duration of 3 min would result in more cell damage compared with 0.5 W/m^2^ and duration of 1 min. Therefore, 0.5 W/m^2^ ultrasound intensity and irradiation duration of 1 min was considered as the optimal transfection condition in our study. The results indicated that ultrasound irradiation combined with NBs carrying siRNA-NBs had a good US-triggered gene transmission ability and were confirmed under flow cytometry (Figures 4(c,d)). Strikingly, as the ultrasound intensity was 0.5 W/m^2^ and the irradiation duration was 1 min, the transfection efficiency was the highest (90.23 ± 1.45%), while the transfection efficiency was 0.77 ± 0.13% in the control group. Therefore, the optimal parameters to obtain the highest transfection efficiency were as follows: ultrasound intensity of 0.5 W/m^2^, irradiation duration of 1 min. These results showed that FITC labeled SCR was successfully transfected into cells. The combination of NBs and US irradiation has distinct potential to improve gene transfection efficiency. NBs combined with ultrasound irradiation have significant potential to improve the efficiency of gene transfection, and have several advantages for NBs’ gene delivery, including the extended duration in blood circulation, low cytotoxicity, limited immunogenicity, and high repeatability (Gao et al., 2019; Xu et al., 2019; Hu et al., 2020; Zhong et al., 2021). The transfection of SCR siRNA with US irradiation and NBs was detected by flow cytometry *in vitro*. The results showed that the transfection efficiency of SCR siRNA-NBs delivery of the SCR siRNA was significantly improved compared with control groups when ultrasound irradiation was added. In conclusion, siRNA-NB has important clinical application value in improving the targeted delivery of siRNA genes (Cai et al., 2015; Zhang et al., 2019).

### 3.7. Suppression of PDLIM5 gene expression in PC9GR cell lines

As shown in Figure 3(d), the inhibition of PDLIM5 mRNA expression in group 7 was more effective compared with other groups (all *p* < .005). Compared with negative control group 1, PDLIM5 mRNA expression in groups 6 and 7 was significantly inhibited (*p* < .01), and that in group 7 was significantly lower than that in group 6 (*p* < .001). Protein expression of PDLIM5 was further detected by Western blotting (Figures 3(e,f)). Similarly, it had the highest inhibition, in group 7 which was consistent with the expression of the PDLIM5 gene. Transfection groups 6 and 7 could effectively inhibit the expression of PDLIM5 protein, and the inhibition of PDLIM5 protein in transfection group 7 was markedly higher than that in any other group (all *p* < .001).

The results of the molecular biological analysis showed that NBs could effectively transfer PDLIM5 siRNA into cells under ultrasound irradiation, leading to the significant downregulation of PDLIM5 protein and mRNA expression. the highest inhibition of PDLIM5 gene expression in NBs plus US groups suggested that ultrasound irradiation and targeting of NBs carrying PDLIM5siRNA synergistically inhibited expression of the PDLIM5 gene.

### 3.8. Cell growth and inhibition assay

**Figure 5.**
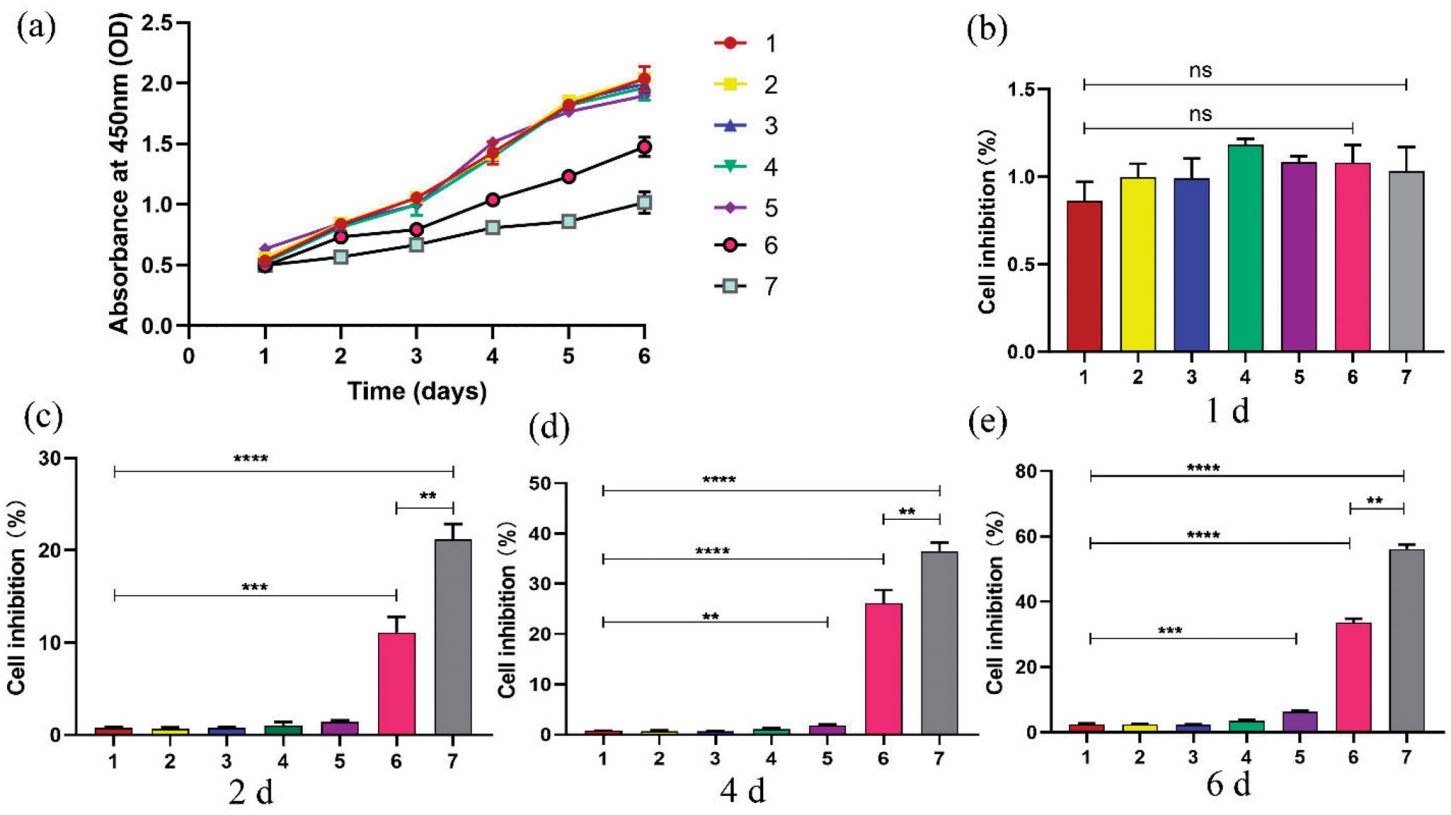
Cell inhibition studies under ultrasound exposure and PDLIM5siRNA-NBs were performed. (a) The growth of cells in group 6 and group 7 was significantly inhibited compared to the other groups, especially in group 7. (b–e) Cell inhibition rates were analyzed after cells were transfected and then incubated at 1, 2, 4, and 6 d in seven groups, Groups 1–7 represent as Table 2. **p < .01, ***p < .005, ****p <.001 Comparison between the two groups.

Ultrasound irradiation is considered a therapeutic method because it can increase the permeability of cell membranes through mechanical stress (Schlicher et al., 2006). Ultrasonic transmission technology is based on the biophysical process of cell pores combined with nanoparticles, which is called ‘sonoporation’ (Karshafian et al., 2009). In recent years, the phenomenon of sonoporation has been studied extensively in enhancing the intracellular transmission of genes (Sakakima et al., 2005; Tu & Zhang, 2017). In this study, the chemotherapy effect of NBs on PC9GR cells was verified by a cell viability study *in vitro*. In addition, the enhancement of the anti-tumor efficiency of NBs by ultrasound irradiation was also studied. The CCK-8 from day 1 to day 6 after transfection showed that the growth of PC9GR cells was inhibited in group 6 and group 7 (Figure 5(a)), and the cell inhibition in group 7 was more significant under the effect of ultrasonic irradiation than that in group 6. On the 6th day after transfection, the growth inhibition rate of PC9GR cells in group 7 was the highest at 56.15 ± 1.36%, followed by group 6 (33.49 ± 1.25%). As shown in Figure 5(b), there was no significant difference in the viability of PC9GR cells intervened with or without ultrasound irradiation at 1d, indicating that PDLIM5siRNA-NBs and PDLIM5siRNA-NBs plus ultrasound irradiation had no cytotoxicity. However, cells that intervened with NBs with or without US irradiation for 2, 4, and 6 d revealed a significant viability decrease, and it was more obvious after ultrasonic irradiation as shown in Figures 5(c–e). The enhanced anticancer effect may be attributed to both the efficient siRNA-mediated active target effect and the increased drug concentration due to the destruction of siRNA-NBs mediated by ultrasound irradiation (Bolin et al., 2016; Lv et al., 2017). In addition, studies have indicated that NBs, together with microflow, microjet, and cavitation of shave wave, would further facilitate the entry of drugs into cells under ultrasound irradiation (Zhu et al., 2016). Therefore, locally released PDLIM5siRNA and increased permeability may be the main reasons for the enhanced anticancer effect.

### 3.9. Apoptosis of PC9GR cells was tested by flow cytometry

**Figure 6.**
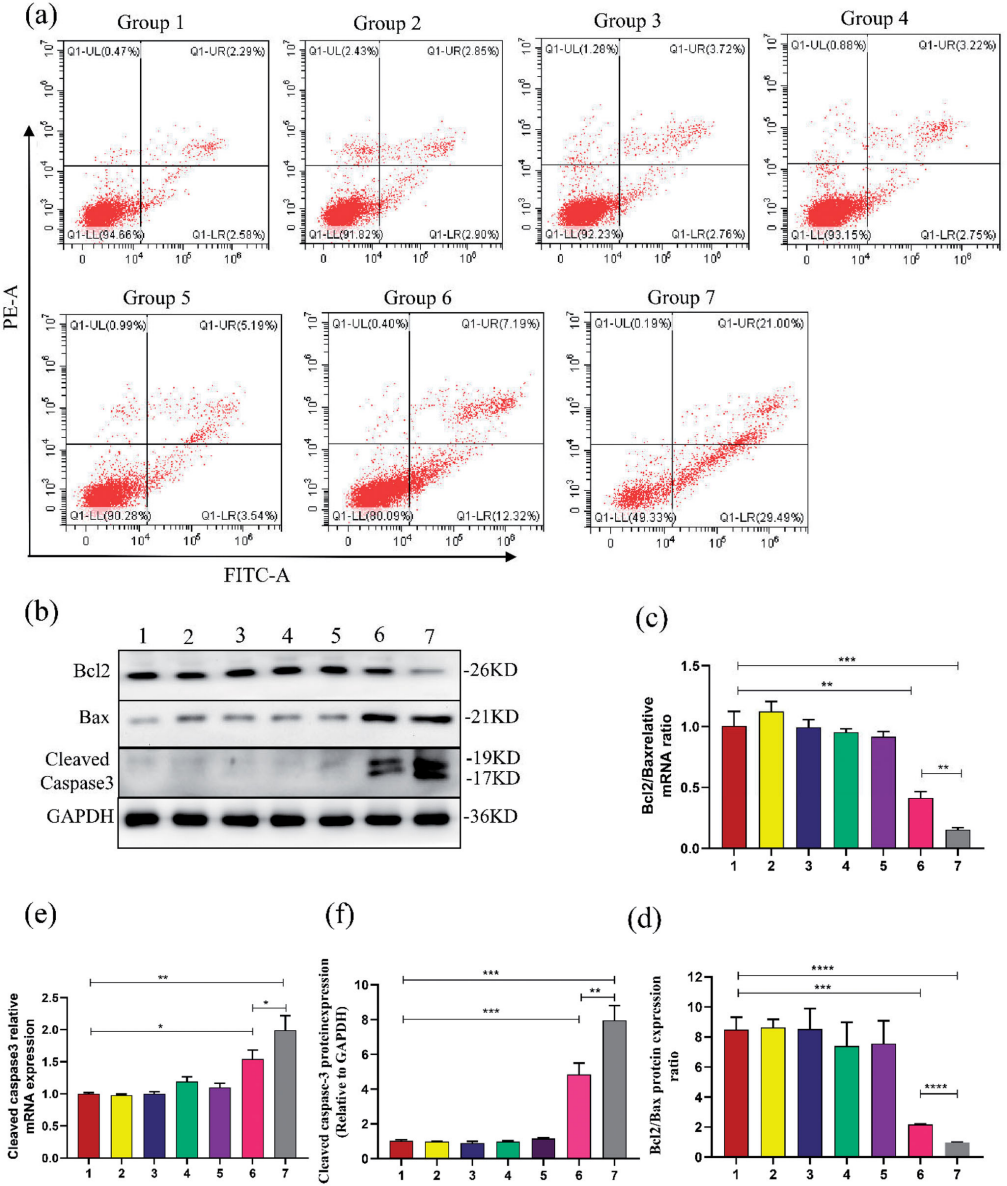
Cell apoptosis analysis was evaluated (a) Cell apoptosis analysis was conducted by flow cytometry to determine the cell apoptosis differences among the seven groups, as shown in Table 2. Q1-UL: necrotic cells; Q1-LL: viable cells; Q1-UR: late apoptotic cells; Q1-LR: early apoptotic cells. (b,d,f) Protein expressions of Bcl2/Bax and Cleaved caspase 3 were detected in PC9GR cells by western blot. (c,e) RT-PCR analysis showed the difference of Bcl2/Bax and Cleaved caspase 3 expressions in PC9GR cells. Groups 1–7 represented as Table 2: (Group 1) Blank, (Group 2) SCR, (Group 3) SCR-NB, (Group 4) PDLIM5siRNA, (Group 5) PDLIM5siRNA + US, (Group 6) PDLIM5siRNA-NB, and (Group 7) PDLIM5siRNA-NB + US. *p < .05, **p < .01, ***p < .005, ****p <.001, Comparison between the two groups.

Further assay of apoptotic cells showed that group 7 had the highest apoptosis rate (50.13 ± 1.29%), followed by group 6 (19.20 ± 0.38%), while group 1 as control almost had no apoptosis (Figure 6(a)). After treatment with US-mediated PDLIM5 siRNA-NBs transfection for 48 h, PC9GR cells apoptosis was significantly increased compared with any other group (*p* < .05). The results indicated that US-mediated PDLIM5 siRNA-NBs transfection can efficiently accelerate NSCLC cells' apoptosis. The improved anticancer effect of PDLIM5siRNA-NBs *in vitro* may be partly attributed to the effective active target effect mediated by PDLIM5siRNA-NBs, and partly to the increased concentration of the drug due to the destruction of PDLIM5siRNA-NBs mediated by the US (Chen et al., 2015). To further determine the molecular mechanism of cell apoptosis, expression of Bcl2/Bax and cleaved Caspase3 was detected by Western blot and RT-PCR (Figures 6(b–f)). The results indicated that after PDLIM5siRNA-NBs intervention promoted cells apoptosis, down-regulated expression of Bcl2/Bax mRNA and protein, and up-regulated expression of Caspase3 mRNA and protein, especially under ultrasound irradiation.

## 4. Conclusion

We devised an ultrasound-sensitive gene delivery strategy for targeted siRNA therapy of drug resistance in NSCLC. With the help of ultrasonic irradiation combined with NBs carrying siRNA, NBs showed the controllable release ability of siRNA to significantly improve the efficiency of siRNA transfection, resulting in strong inhibition of the expression of the PDLIM5 gene in PC9GR cells. After the successful delivery of siRNA, cell proliferation was prevented, and apoptosis was promoted with no significant cytotoxicity. It showed significant anticancer efficacy *in vitro*. Our results suggest that NBs carrying siRNA combined with the US may be a promising strategy for ultrasonic-triggered PDLIM5 siRNA delivery for targeting genes and chemotherapy.
